# Multi-scale ecosystem service dataset for Southern Hilly Region of China (1990–2024)

**DOI:** 10.1038/s41597-026-07942-5

**Published:** 2026-07-25

**Authors:** Zhiying Tang, Yaoxing Wu, Chen Ding, Hanqing Song, Lianghua Qi

**Affiliations:** 1 International Center for Bamboo and Rattan, Beijing, 100102 China; 2 Georgina Mace Centre for the Living Planet, Department of Life Sciences, Imperial College London, Silwood Park, Ascot, Berkshire, SL5 7PY UK; 3 Beijing Changping “Huitian” Area Parks & Green Space Management Center, Beijing, 102200 China

## Abstract

Ecosystem services are spatially heterogeneous and scale dependent, which makes long-term and cross-scale datasets essential for regional ecological assessment and environmental management. However, for the Southern Hilly Region of China (SHR), ecosystem service datasets that are temporally explicit, methodologically consistent, and organized across multiple watershed-based management scales remain limited. Here, we present a spatially explicit, multi-scale ecosystem service dataset for the SHR covering eight temporal snapshots from 1990, 1995, 2000, 2005, 2010, 2015, 2020 to 2024 at a base spatial resolution of 30 m. The dataset quantifies five key ecosystem services, namely water yield, nutrient delivery ratio, soil conservation, carbon storage, and habitat quality, using a unified workflow based on InVEST version 3.14.1. Ecosystem services were first generated as raster outputs and were subsequently aggregated to watershed and sub-watershed units within a consistent hierarchical spatial framework. Compared with existing ecosystem service products that are often national or global in scope, coarser in temporal detail, or not organized for watershed-based multi-scale analysis, this dataset provides a region-specific, high-resolution, and reusable product for long-term ecosystem service assessment in the SHR. All input layers were harmonized to a common spatial framework, annual land-cover and climate inputs were matched to the corresponding model years, and static soil, topographic, carbon-density, and habitat-sensitivity parameters were kept constant to support temporal comparability. The dataset has been deposited in Zenodo under the Creative Commons Attribution 4.0 International license and is available at 10.5281/zenodo.18329300^1^.

## Background & Summary

Ecosystem services refer to the direct and indirect benefits that natural ecosystems provide to human society through their structures, processes, and functions, forming a fundamental basis for human well-being, ecological security, and sustainable development^2,3^. With increasing intensity of human activities, land use and land cover change has substantially altered ecosystem structure and functioning, leading to pronounced changes in magnitude and spatial distribution of ecosystem services^4^.

A growing body of research has demonstrated that ecosystem services exhibit strong spatial heterogeneity and temporal variability, and that their dominant processes and spatial patterns differ markedly across spatial scales. Such scale dependency poses challenges for ecosystem service assessment, comparison, and application in environmental management and policy-making contexts^5,6^.

The Southern Hilly Region of China (SHR) represents one of the country’s most important ecological security barriers and agricultural production regions^7^. Characterized by complex topography, a humid subtropical monsoon climate, and high biodiversity, the region has long been subject to pressures from soil erosion, habitat fragmentation, and rapid land-use change^8,9^. In recent years, large-scale ecological protection and restoration initiatives have further emphasized the strategic importance of this region for regional sustainability and ecological governance^10^.

Despite extensive research on ecosystem services in southern China, most existing studies and data products remain limited in at least one of three aspects. First, many studies focus on single administrative regions, individual watersheds, or single time periods, which restricts long-term comparison. Second, existing ecosystem service products are often generated at national or global scales and are not specifically designed for the complex terrain, humid subtropical climate, and watershed-based management needs of the SHR. Third, few datasets provide both raster outputs and spatially aggregated attributes across regional, watershed, and sub-watershed scales using the same model configuration and spatial framework^6,11^. The main contribution of this Data Descriptor is therefore not the development of a new ecosystem service model, but the construction of a harmonized, reproducible, and spatially nested dataset that integrates high-resolution input data, standardized InVEST modelling, and hierarchical spatial aggregation for the SHR.

By combining 30 m land-cover information, climate, soil, topographic, carbon-density, and habitat-related parameters within a consistent modelling workflow, the dataset supports broad-scale regional assessment and finer-scale watershed applications. It is designed to help users examine spatial patterns, temporal changes, and scale-dependent differences in ecosystem service variables across the SHR, while recognizing that the data are model-derived products rather than direct field observations.

## Methods

### Study area and spatial framework

**Fig. 1:**
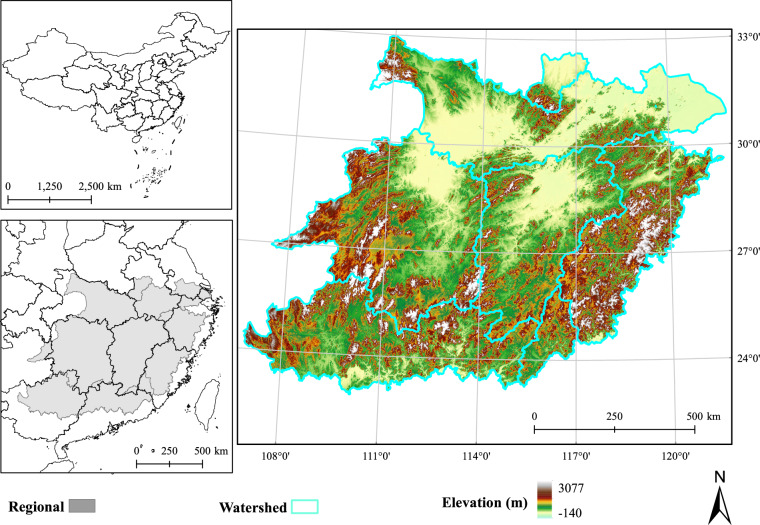
Geographical location and overview of the study area.

The Southern Hilly Region of China (Fig. 1) is located south of the Yangtze River and north of the Pearl River, extending approximately from 22°50′N to 32°00′N and from 106°50′E to 122°00′E. In this study, the SHR boundary followed the spatial extent of the southern hilly and mountainous region defined in the national ecological protection and restoration planning framework, and the same SHR boundary layer has been used in related work on biodiversity conservation in this region^7,10^. The boundary was therefore treated as an established ecological-management region rather than as a newly classified geomorphological zone. For reproducibility, the exact SHR polygon used in this study is provided in the released vector dataset and was used as the clipping mask for all raster inputs and outputs. The region encompasses parts of Anhui, Fujian, Guangdong, Guangxi, Guizhou, Hubei, Hunan, Jiangsu, Jiangxi, Shanghai, and Zhejiang provinces and municipalities, covering approximately 0.977 million km^2^. For multi-scale calculations, the study area was organized into a hierarchical spatial framework consisting of the whole SHR, 15 major watershed units, and 4522 sub-watershed units. The region is dominated by low mountains, hills, and tablelands and is characterized by a humid subtropical monsoon climate with abundant precipitation and diverse vegetation types. Based on digital elevation model (DEM)-derived hydrological analysis, watershed and sub-watershed boundaries were delineated from a depression-filled and peak-trimmed DEM by calculating flow direction and accumulation, extracting river networks using a threshold method, and identifying basin outlets.

The dataset adopts a spatio-temporal structure comprising regional, watershed, and sub-watershed spatial scales, and eight temporal snapshots (1990, 1995, 2000, 2005, 2010, 2015, 2020 and 2024).

### Input data processing

Multiple input datasets were compiled for ecosystem service modelling, including land use/land cover (LULC), precipitation, potential evapotranspiration, soil properties, topographic data, watershed boundary layers, biophysical tables, carbon-density parameters, and habitat-related threat and sensitivity information. To improve transparency and reproducibility, the raw data sources, spatial resolutions, temporal coverage, main variables, and preprocessing procedures are summarized in Table 1, while detailed module-specific parameters are provided in the Supplementary Data Input Checklist. All third-party input datasets were obtained from publicly accessible data repositories, institutional download portals, or published literature; the corresponding DOI, URL, or bibliographic source is provided in Table 1, the Methods, and the Supplementary Data Input Checklist. The released Zenodo repository contains the derived ecosystem service products and documentation rather than redistributing third-party raw input datasets. Annual land-cover and climate layers were extracted or aggregated for the corresponding model years and then matched within the same temporal snapshot. Static or slowly varying layers, including DEM-derived terrain information, watershed boundaries, soil attributes, depth to bedrock, carbon-density coefficients, and habitat-sensitivity parameters, were held constant across the modelling years to maintain temporal comparability. All raster datasets were reprojected to a unified projected coordinate reference system, clipped to the SHR boundary, and aligned to a common 30 m grid. Continuous variables were resampled using bilinear interpolation where necessary, whereas categorical variables were resampled using the nearest-neighbour method. LULC classes were harmonized into six categories using the final codebook shown in Table 2.

**Table 1:** Main input datasets used for InVEST ecosystem service modelling.

Input dataset	Source/product	Native spatial resolution	Main preprocessing procedures
Land use/land cover (LULC)	China 30 m annual land-cover dataset^21^	Raster (.tif), 30 m	Temporal snapshots (1990, 1995, 2000, 2005, 2010, 2015, 2020 and 2024) were extracted for the selected model years and reclassified according to the final LULC codebook in Table 2. The same class codes were used in all InVEST lookup tables.
Annual precipitation	1 km monthly precipitation dataset for China (1901–2024)^22^	Raster (.tif), 1 km	Monthly precipitation was aggregated to annual precipitation for each model year, clipped to the SHR boundary, resampled and aligned to the 30 m modelling grid, and used in the water yield module and as the basis for rainfall in the SDR workflow.
Potential evapotranspiration	1 km monthly potential evapotranspiration dataset for China (1901–2024)^23^	Raster (.tif), 1 km	Monthly potential evapotranspiration was aggregated to annual potential evapotranspiration for each model year, clipped to the SHR boundary, resampled to the common 30 m grid, and matched with precipitation and LULC layers.
Root restricting layer depth and plant available water content	Depth-to-bedrock map of China^24^, HWSD v2.0 soil attributes^25^; PAWC estimation followed Zhou *et al*.^26^	Raster (.tif), 100 m for depth to bedrock; soil data resampled to model grid	Depth-to-bedrock was used as a proxy for root restricting layer depth and converted from metres to millimetres following InVEST input requirements. PAWC was derived from soil texture, gravel fraction, clay/silt content and organic carbon, and then projected, clipped, resampled, and aligned with other inputs.
Digital elevation model (DEM) and hydrological boundaries	DEM from Geospatial Data Cloud; watershed delineation followed DEM-based hydrological analysis procedures^27,28^.	Raster (.tif) and vector (.shp)	DEM preprocessing included sink filling, slope calculation, flow direction, flow accumulation, stream extraction and outlet identification. Watershed and sub-watershed boundaries were generated from the processed DEM and used for routing and zonal aggregation.

Note: detailed parameter values and module-specific input assumptions are provided in the Supplementary Data Input Checklist.

**Table 2:** Final LULC codebook used for all InVEST model runs.

Code	LULC class	Description in released data
1	Cropland	Cultivated land and other agricultural land
2	Forest	Forest land, shrubland and other tree-dominated vegetation
3	Grassland	Grassland and herbaceous vegetation
4	Water area	Rivers, lakes, reservoirs and other water bodies
5	Impervious	Urban, built-up and other impervious surface
6	Barren	Bare land and sparsely vegetated unused land

### Ecosystem service modelling

The final LULC codebook used in all InVEST lookup tables was fixed before model execution. The same codes were used for the WY, NDR, SC, CS and HQ modules to avoid inconsistencies among biophysical tables.

Five ecosystem services were quantified in this dataset: Water yield (WY), Nutrient delivery ratio (NDR), Soil conservation (SC), Carbon storage (CS), Habitat quality (HQ).

All ecosystem services were modelled using InVEST version 3.14.1^12,13^, following a unified modelling framework. Ecosystem services were first calculated at the raster scale with a spatial resolution of 30 m, and the same model version, LULC codebook, static parameters, and spatial processing rules were applied across the temporal snapshots. The principal released variables and aggregation rules are listed in Table 3. WY represents annual water yield depth from the InVEST water yield module. The NDR-related released product represents nutrient export generated by the InVEST Nutrient Delivery Ratio module; it should therefore be interpreted as a nutrient export variable rather than as a retention-efficiency coefficient. SC represents avoided erosion derived from the SDR workflow, defined as the difference between potential soil loss and actual soil loss under observed land cover and management parameters. CS represents carbon stock estimated from the four InVEST carbon pools. HQ represents the dimensionless habitat-quality score from the InVEST habitat quality module. Raster outputs were subsequently aggregated to watershed and sub-watershed scales using zonal statistics. Area-weighted means were reported for intensity or index variables, while zonal totals were documented where the variable represented a stock or total amount.

**Table 3:** Released ecosystem service variables and units.

Service	Released raster variable	Unit/scale
WY	Annual water yield depth from the InVEST water yield module	mm/yr
NDR	Nutrient export output from the InVEST NDR module	kg/ha/yr
SC	Avoided erosion from the InVEST SDR module, defined as potential soil loss minus actual soil loss	t/ha/yr
CS	Carbon stock from aboveground, belowground, soil and dead organic matter pools	Mg∙C
HQ	Habitat-quality score from the InVEST habitat quality module	Dimensionless, 0–1

Module-specific parameter values, units, and sources are documented in the Supplementary Data Input Checklist, including the water yield biophysical table and Z parameter, nutrient export-related coefficient for the NDR module, SDR cover-management and support-practice factors, carbon-pool density values, habitat threat and sensitivity parameters, and the half-saturation constant. Where literature-based parameterization was required, previously published studies in comparable Chinese watershed or hilly-region contexts were used as references. For example, nutrient export and water-purification related parameterization was informed by InVEST applications and nutrient-loss studies in southern Chinese watersheds and reservoir areas^14,15^. Soil erosion and soil conservation related parameters were checked against published USLE- and InVEST-based studies on soil erosion assessment in China and low-hilly areas^16–18^. This structure keeps the main text focused on dataset design and interpretation while retaining the information needed for model reuse and reproducibility.

### Data characteristics

To improve the ready-to-use value of the dataset, this section provides representative visualizations and a concise overview of the main temporal and spatial characteristics contained in the released data. The purpose of this section is descriptive: it helps users understand the structure, temporal summaries, and broad spatial gradients of the dataset before reuse, while detailed process attribution and hypothesis-driven analysis are beyond the scope of this Data Descriptor.

**Fig. 2:**
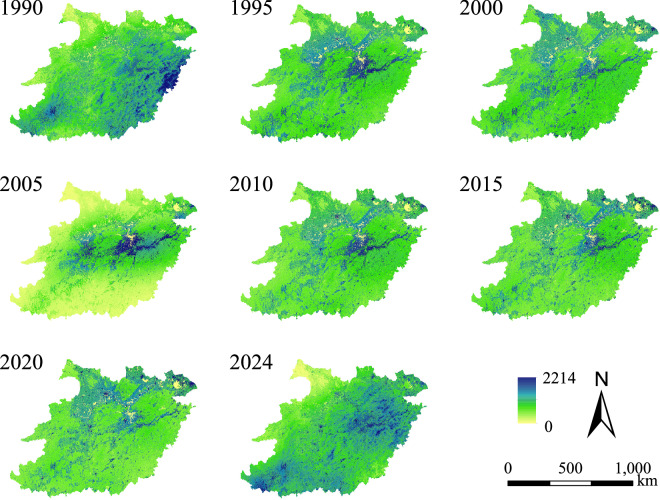
Spatial distribution of WY in representative years.

**Fig. 3:**
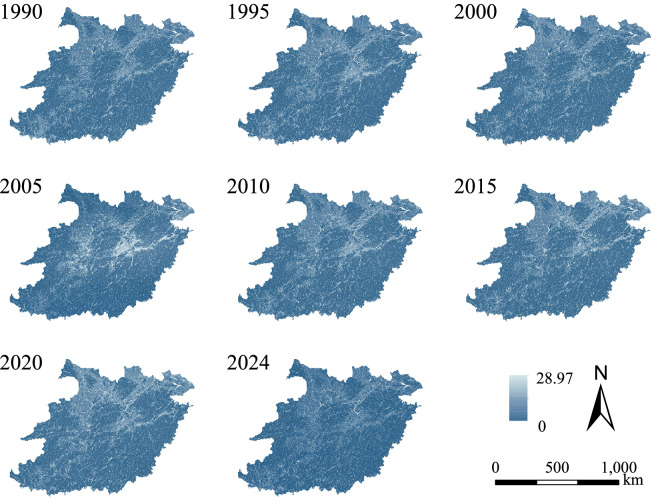
Spatial distribution of NDR in representative years.

**Fig. 4:**
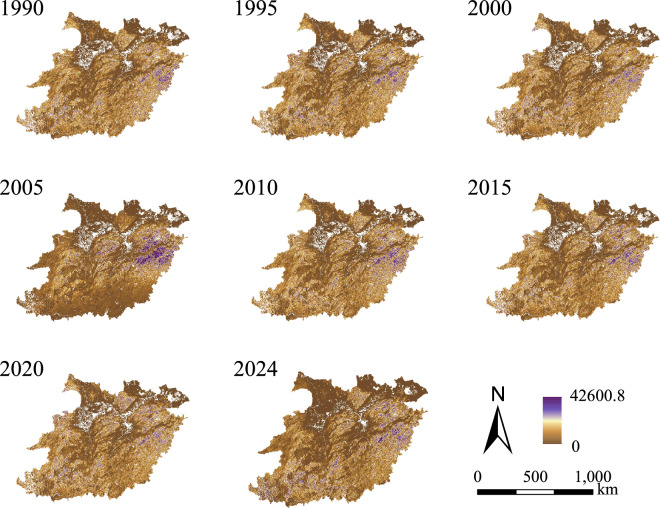
Spatial distribution of SC in representative years.

**Fig. 5:**
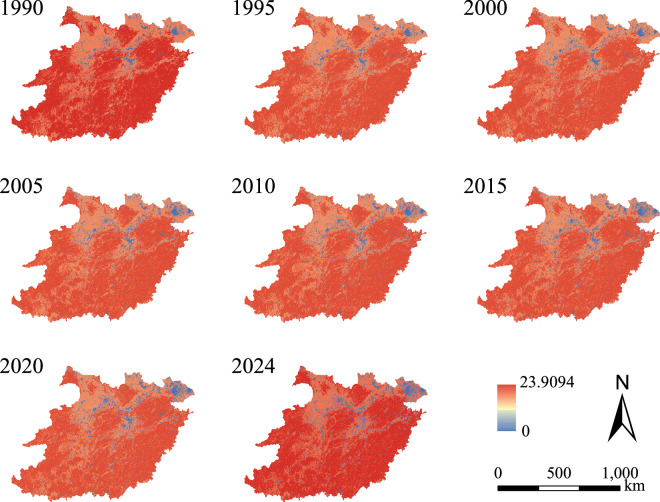
Spatial distribution of CS in representative years.

**Fig. 6:**
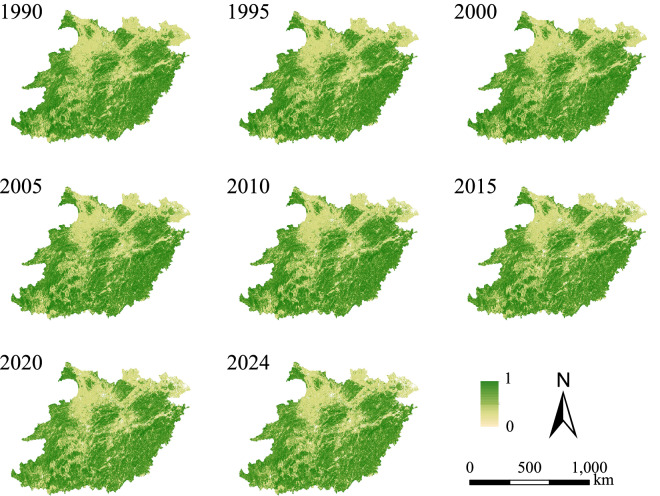
Spatial distribution of HQ in representative years.

The spatial distributions of the five released ecosystem service variables are shown in Figs. 2–6. WY high-value areas are mainly associated with mountainous and hilly areas such as the Wuyi Mountains, Nanling Mountains, and surrounding uplands. SC and HQ also tend to be higher in forested mountain and hill regions, whereas lower values are commonly found in intensively cultivated plains, urbanized areas, and river-valley or coastal lowlands. CS shows a relatively stable spatial pattern linked to the distribution of persistent forest cover. These spatial gradients are consistent with the climate, topographic, and land-cover structure of the SHR. The eight temporal snapshots further allow users to compare ecosystem service changes across more than three decades under a consistent spatial framework, but detailed process attribution and analysis should be conducted in subsequent analytical studies rather than treated as the primary aim of this Data Descriptor. These visual summaries are intended to guide reuse of the dataset rather than to provide a full ecological interpretation of the observed patterns.

## Data Records

The dataset has been deposited in the Zenodo data repository and is publicly available through the formal data citation listed in Ref. ^1^ at https://zenodo.org/records/18329300. All data are provided in commonly used geospatial and tabular formats to facilitate reuse in geographic information systems and statistical analysis platforms.

Raster data consist of spatially explicit ecosystem service outputs for WY, NDR, SC, CS and HQ. Raster datasets are provided in GeoTIFF format at a spatial resolution of 30 m for each temporal snapshot and are organized by ecosystem-service type and year. The released variable represented by each raster is defined in Table 3, and the full file naming convention and units are provided in the data documentation package.

Vector data include polygon boundary layers for the study region, major watershed units, and sub-watershed units. These datasets are provided in ESRI Shapefile format and serve as spatial reference layers for aggregation and analysis.

The accompanying documentation package includes file organization, naming conventions, variable definitions, units, coordinate reference systems, and processing steps.

A detailed Supplementary Data Input Checklist documents the InVEST model input datasets, parameter values, data sources, and preprocessing assumptions to facilitate reproducibility.

## Technical Validation

The technical validation focused on the traceability, internal consistency, and reproducibility of the input data and model configuration. Because field observations covering all five ecosystem services, all temporal snapshots, and the entire SHR were not available, the dataset was not evaluated as a site-level observation product. The validation was therefore designed as a technical and consistency assessment of a model-derived geospatial dataset. This treatment is consistent with previous discussions of ecosystem service model validation and uncertainty, which emphasize that model-derived ecosystem service outputs should be interpreted with attention to model structure, parameter assumptions, and data availability rather than treated as direct observations^19,20^.

Before model implementation, all input datasets were organized according to the requirements of the corresponding InVEST modules. Raster datasets were checked for coordinate reference system, spatial extent, spatial resolution, pixel alignment, unit consistency, and correspondence between LULC class codes and lookup tables. The main preprocessing steps included projection transformation, clipping to the SHR boundary, unit conversion where required, resampling or alignment to the common modelling grid, and matching of temporal snapshots with the corresponding land-cover and climate inputs.

Module-level checks were then conducted before model execution. For WY, precipitation, potential evapotranspiration, root-restricting layer depth, plant available water content, LULC, watershed boundaries, and the biophysical table were checked for format and unit consistency. For NDR, the DEM, LULC, nutrient runoff proxy, watershed boundaries, and nutrient-related biophysical parameters were prepared following the InVEST input structure. For SDR/SC, DEM-derived terrain inputs, rainfall-related inputs, soil erodibility, LULC, USLE parameters, watershed boundaries, and routing parameters were compiled and checked. For CS, each LULC class was matched with the four carbon-pool density parameters. For HQ, LULC, threat layers, sensitivity values, and the half-saturation constant were organized according to the InVEST habitat quality requirements.

The spatial aggregation framework was checked by using the same SHR boundary, watershed units, and sub-watershed units throughout the workflow. Watershed and sub-watershed boundaries were generated from DEM-based hydrological analysis and were used consistently for organizing raster outputs and producing vector-based zonal summaries. This procedure ensured that raster outputs and aggregated attributes were spatially nested within the same framework.

The main limitations of the validation are also documented to support appropriate reuse. The dataset is derived from ecosystem service models and should not be interpreted as direct field measurements. Several input layers, such as climate and soil data, have coarser native resolutions than the final 30 m modelling grid, which may introduce uncertainty after resampling. Some parameters were derived from literature values, local documents, expert judgement, or proxy variables, and their transferability may vary across the SHR. In addition, rainfall-related inputs used in the SDR workflow cannot fully represent sub-daily rainfall intensity or extreme rainfall events. These limitations should be considered when absolute values are interpreted or when the dataset is used for site-specific management decisions.

Accordingly, this dataset should be regarded as a harmonized, model-derived, and reproducible ecosystem service data product for regional-scale, watershed-scale and sub-watershed-scale analysis. It is suitable for comparative assessment of spatial patterns, temporal changes, and multi-scale differences in ecosystem services, while future updates could further strengthen validation by incorporating observed streamflow, sediment monitoring, water-quality data, forest inventory or biomass products, and biodiversity observation records where such data become available.

## Usage Notes

This dataset provides a comprehensive representation of the spatial distribution and temporal evolution of ecosystem services in the SHR from 1990 to 2024. The multi-scale structure enables analyses ranging from regional-scale assessments to watershed- and sub-watershed-level investigations.

The dataset is suitable for applications such as ecosystem service assessment, watershed management, ecological restoration evaluation, degradation diagnosis, restoration-priority identification, and multi-scale comparative studies. It can support academic research and policy-oriented applications, including assessment of ecological protection and restoration projects, analysis of climate and human-activity drivers, and evidence-based management of mountain-water-forest-farmland-lake-grassland-sand systems in the SHR. Users should note that the dataset is derived from spatially explicit ecosystem service models and is not intended to replace site-specific field measurements. Uncertainties associated with input data quality, spatial resolution, parameter assumptions, and model structure should be considered when interpreting results.

## Supplementary information

parameter

Supplementary_Data

## Data Availability

The complete multi-scale ecosystem service dataset for the Southern Hilly Region of China (1990–2024) is publicly available via Zenodo^1^. The repository contains raster outputs (GeoTIFF, 30 m) for five ecosystem service variables across eight temporal snapshots, boundary layers (ESRI Shapefile) for the regional/watershed/sub-watershed spatial framework, and documentation describing file structure, variable definitions, units, and coordinate reference system. All files can be accessed anonymously without login. The dataset is distributed under the Creative Commons Attribution 4.0 International license.

## Code availability

No custom code was used to generate the dataset. Ecosystem service modelling was performed using InVEST version 3.14.1, and geospatial preprocessing, raster alignment, and zonal statistics were performed using standard GIS tools as described in the Methods. Existing third-party tools and datasets are cited in the Methods and References.
