# Supplementary material for: Multi-scale ecosystem service dataset for Southern Hilly Region of China (1990–2024)
Source: Sci Data. 2026 Jul 25;13:1133. doi: 10.1038/s41597-026-07942-5 (PMC13443169; doi:10.1038/s41597-026-07942-5)
Supplement: Supplementary file 1 [file 41597_2026_7942_MOESM2_ESM.docx]

This document provides a comprehensive checklist of all input datasets, parameters, and preprocessing steps used in the InVEST ecosystem service models described in this Data Descriptor. It is intended to support transparency, reproducibility, and reuse of the dataset.

**InVEST Model Data Input Checklist**

**General Prerequisites:**

- **Spatial Alignment:** All raster datasets **must** have the **same spatial resolution**, the same spatial extent, and be perfectly aligned.
- **Coordinate Reference System:** All datasets must be in the same projected coordinate system.
- **Land Use/Land Cover (LULC) Map:** This is a core input for most models. The class codes and names in the LULC raster must exactly match those used in all related lookup tables.

**1. Annual Water Yield**

- **Annual Precipitation**
- **Description**: Annual precipitation (mm/yr) for the corresponding modelling years within 1990-2024 was aggregated from monthly data and extracted for the study area using a mask.
- **Sources:** Peng, S. (2020). 1-km monthly precipitation dataset for China (1901-2024). National Tibetan Plateau / Third Pole Environment Data Center. <https://doi.org/10.5281/zenodo.3114194>.
- **Format:** Raster (.tif)
- **Evapotranspiration (ET₀)**
- **Description**: Annual evapotranspiration (mm/yr) for the corresponding modelling years within 1990-2024 was aggregated from monthly data and extracted for the study area using a mask.
- **Sources:** Peng, S. (2022). 1-km monthly potential evapotranspiration dataset for China (1901-2024). National Tibetan Plateau / Third Pole Environment Data Center. <https://doi.org/10.11866/db.loess.2021.001>.
- **Format:** Raster (.tif)
- **Root Restricting Layer Depth**
- **Description**: Use Bedrock depth instead (Yan et al., 2020). Multiply by 1000 to convert meters into millimeters (Natural Capital Project, 2024).
- **Sources:** Yan, F., Shangguan, W., Zhang, J. et al. Depth-to-bedrock map of China at a spatial resolution of 100 meters. Sci Data 7, 2 (2020). <https://doi.org/10.1038/s41597-019-0345-6>
- **Format:** Raster (.tif)
- **Plant Available Water Content**
- **Description**: The proportion of gravel, silt, clay particles and organic carbon in the 0-200 cm soil layer is calculated (Zhou et al., 2005).
- **Sources:** FAO & IIASA. 2023. Harmonized World Soil Database version 2.0. Rome and Laxenburg. <https://doi.org/10.4060/cc3823en>
- **Format:** Raster (.tif)
- **Land Use/Land Cover (LULC)**
- **Description**: Raster where each pixel value represents a land-cover class. The final codebook must be used consistently across all model lookup tables: 1=cropland, 2=forest, 3=grassland, 4=water area, 5=impervious, 6=barren, unless the authors confirm a different final codebook and update every table accordingly.
- **Sources:** Jie Yang, & Xin Huang. (2025). The 30 m annual land cover datasets and its dynamics in China from 1985 to 2024 [Data set]. <https://doi.org/10.5281/zenodo.12779975>
- **Format:** Raster (.tif)
- **Biophysical Table**

**Description**: - Kc Parameter: Crop/vegetation coefficient (unitless), varies by vegetation type (Chen et al., 2021).

| description | lucode | root_depth | Kc | LULC_veg |
| --- | --- | --- | --- | --- |
| Cropland | 1 | 2000 | 0.65 | 1 |
| Forest | 2 | 5200 | 1 | 1 |
| Grass land | 3 | 2500 | 0.85 | 1 |
| Water area | 4 | 100 | 1 | 0 |
| Impervious | 5 | 100 | 0.3 | 0 |
| Barren | 6 | 300 | 0.2 | 0 |

- **Sources:** Literature review (Chen et al., 2021).
- **Format:** Table (.csv)
- **Z Parameter**
- **Description**: Empirical constant related to root depth and soil properties. After comparison and verification with the China Water Resources Bulletin, the input parameters were adjusted.
- **Sources:** Local experimental data.
- **Format:** Number
- **Watershed/Sub-Watershed**
- **Description**: Watershed boundaries are delineated from a depression-filled and peak-trimmed DEM by calculating flow direction and accumulation, extracting a river network using a threshold method, and identifying the basin outlet (Xu et al., 2004; Zhang et al., 2010).
- **Sources:** DEM: <https://www.gscloud.cn/>
- **Format:** Vector (.shp)
- **Water Demand Table** (Optional)
- **Description**: A table of water demand for each LULC class. Each LULC code in the LULC raster must have a corresponding row in this table.
- **Sources:** Literature review
- **Format:** Table (.csv)
- **Hydropower Valuation Table** (Optional)
- **Description**: A table mapping each watershed to the associated valuation parameters for its hydropower station.
- **Sources:** OpenStreetMap (<https://www.openstreetmap.org/>), local water authority data.
- **Format:** Table (.csv)

**2. Nutrient & Sediment Delivery Ratio**

- **Digital Elevation Model (DEM)**
- **Sources:** <https://www.gscloud.cn/>
- **Format:** Raster (.tif)
- **Land Use/Land Cover (LULC)**
- **Description**: Raster where each pixel value represents a land-cover class. The final codebook must be used consistently across all model lookup tables: 1=cropland, 2=forest, 3=grassland, 4=water area, 5=impervious, 6=barren, unless the authors confirm a different final codebook and update every table accordingly.
- **Sources:** Jie Yang, & Xin Huang. (2025). The 30 m annual land cover datasets and its dynamics in China from 1985 to 2024 [Data set].. <https://doi.org/10.5281/zenodo.12779975>
- **Nutrient Runoff Proxy**
- **Description**: Used for effective nutrient load
- **Sources:** Annual Water Yield output
- **Format:** Raster (.tif)
- **Watersheds**
- **Description**: Watershed boundaries are delineated from a depression-filled and peak-trimmed DEM by calculating flow direction and accumulation, extracting a river network using a threshold method, and identifying the basin outlet (Xu et al., 2004; Zhang et al., 2010).
- **Sources:** DEM: <https://www.gscloud.cn/>
- **Format:** Vector (.shp)
- **Biophysical Table**
- **Description**:

- Load_N and Load_P are effective nutrient load/export coefficients used by land-cover class; the sediment-related parameter is reported separately and should not be interpreted as a nutrient retention coefficient.

- Sediment retention parameters are expressed as percentages where applicable and should be distinguished from 0-1 nutrient retention efficiencies.

- **Sources:** Literature values (Han et al., 2016), local monitoring data.

| LU Type | Load_N | Load_P | Sediment retention parameter (%) |
| --- | --- | --- | --- |
| Cropland | 7.71 | 4.68 | 25 |
| Forest | 1.8 | 0.011 | 70 |
| Grassland | 3.1 | 0.1 | 48 |
| Water area | 0.001 | 0.001 | 5 |
| Impervious | 11 | 0.24 | 5 |
| Barren | 14.9 | 0.51 | 5 |

- **Format:** Table (.csv)
- **Threshold Flow Accumulation**
- **Description**: The number of upslope pixels that must flow into a pixel before it is classified as a stream.
- **Sources:** Literature values (Zhang et al., 2016), local monitoring data.
- **Format:** Number
- **Borselli K Parameter**
- **Description**:

- Load_N and Load_P are effective nutrient load/export coefficients used by land-cover class; the sediment-related parameter is reported separately and should not be interpreted as a nutrient retention coefficient.

- - Sediment retention parameters are expressed as percentages where applicable and should be distinguished from 0-1 nutrient retention efficiencies.**Sources:** Literature values (Zhang et al., 2016), local monitoring data.
- **Format:** Number

**3. Carbon Storage & Sequestration**

- **Current LULC**
- **Description**: Raster where each pixel value represents a land-cover class. The final codebook must be used consistently across all model lookup tables: 1=cropland, 2=forest, 3=grassland, 4=water area, 5=impervious, 6=barren, unless the authors confirm a different final codebook and update every table accordingly.
- **Sources:** Jie Yang, & Xin Huang. (2025). The 30 m annual land cover datasets and its dynamics in China from 1985 to 2024 [Data set]. <https://doi.org/10.5281/zenodo.12779975>
- **Format:** Raster (.tif)
- **Carbon Pools**
- **Description**: A core CSV table specifying the carbon density for each LULC class across four pools:
- - C_above: Aboveground biomass carbon (tC/ha)
- - C_below: Belowground biomass carbon (tC/ha)
- - C_soil: Soil organic carbon (tC/ha)
- - C_dead: Dead organic matter/litter carbon (tC/ha)
- **Sources:** Local forest inventory or soil survey data.

| Lucode | LULC_Name | C_above | C_below | C_soil | C_dead |
| --- | --- | --- | --- | --- | --- |
| 1 | cropland | 46.5 | 80.7 | 108.4 | 1 |
| 2 | forest | 20.36 | 67.5 | 170 | 7.8 |
| 3 | grassland | 4.3 | 86.5 | 89.02 | 0 |
| 4 | water area | 0 | 0 | 0 | 0 |
| 5 | impervious | 0.74 | 0.13 | 69.92 | 0 |
| 6 | barren | 0 | 0 | 71 | 0 |

- **Format:** Table (.csv)

**4. Habitat Quality Model**

- **Current Land Cover**
- **Description**: Raster where each pixel value represents a land-cover class. The final codebook must be used consistently across all model lookup tables: 1=cropland, 2=forest, 3=grassland, 4=water area, 5=impervious, 6=barren, unless the authors confirm a different final codebook and update every table accordingly.
- **Sources:** Jie Yang, & Xin Huang. (2025). The 30 m annual land cover datasets and its dynamics in China from 1985 to 2024 [Data set].<https://doi.org/10.5281/zenodo.12779975>
- **Format:** Raster (.tif)
- **Threats Table**
- **Description**: A CSV table defining the characteristics of each threat:

- MAX_DIST: The maximum distance (in meters/km) over which the threat exerts an influence.

- WEIGHT: The relative impact of the threat (0-1).

- DECAY: The type of decay with distance (linear or exponential).

- **Sources:** Expert opinion, literature (Zhang et al., 2020), Local land use planning documents.
- **Format:** Table (.csv)
- **Sensitivity Table**
- **Description**: A CSV table specifying the sensitivity (0-1) of each habitat type (LULC class) to each threat. A value of 1 indicates high sensitivity.
- **Sources:** Expert opinion, literature (Zhang et al., 2020), species habitat requirement analysis (Tang et al., 2025).

| LULC | NAME | HABITAT | crop | barren | impervious |
| --- | --- | --- | --- | --- | --- |
| 0 | nodata | 0 | 0 | 0 | 0 |
| 1 | cropland | 0.3 | 0 | 0.4 | 0.8 |
| 2 | forest | 1 | 0.6 | 0.2 | 0.4 |
| 3 | grassland | 1 | 0.8 | 0.6 | 0.6 |
| 4 | water area | 0.7 | 0.5 | 0.2 | 0.4 |
| 5 | barren | 0.6 | 0 | 0.1 | 0 |
| 6 | impervious | 0 | 0.6 | 0 | 0.4 |

- **Format:** Table (.csv)
- **Half-Saturation Constant**
- **Description**: A key model parameter that shapes the response of habitat quality to threat levels. Should be calibrated with field data on species richness if possible.
- **Sources:** Literature (common starting value is 0.5), then fitted using biodiversity observation data.
- **Format:** Number

**5. Sediment Delivery Ratio**

- **Digital Elevation Model (DEM)**
- **Sources:** <https://www.gscloud.cn/>
- **Format:** Raster (.tif)
- **Erosivity**
- **Description**: Rainfall-related input from annual precipitation. This input reflects rainfall-driven erosive potential at the annual scale and should be interpreted as an empirical rainfall-related proxy rather than direct sub-daily rainfall erosivity.
- **Sources:** Peng S. (2020). Monthly Precipitation Dataset with 1km Resolution in China (1901-2022). National data center qinghai-tibet plateau. <https://doi.org/10.5281/zenodo.3185722>
- **Format:** Raster (.tif)
- **Soil Erodibility**
- **Description**: It refers to the ease with which soil particles are separated and transported by water force, mainly related to soil physical and chemical properties such as soil texture, organic matter content, soil structure, and permeability.
- **Sources:** Literature (Han et al., 2022).
- **Format:** Raster (.tif)
- **Land Use/Land Cover**
- **Description**: Raster where each pixel value represents a land-cover class. The final codebook must be used consistently across all model lookup tables: 1=cropland, 2=forest, 3=grassland, 4=water area, 5=impervious, 6=barren, unless the authors confirm a different final codebook and update every table accordingly.
- **Sources:** Jie Yang, & Xin Huang. (2025). The 30 m annual land cover datasets and its dynamics in China from 1985 to 2024 [Data set].<https://doi.org/10.5281/zenodo.12779975>
- **Format:** Raster (.tif)
- **Biophysical Table**
- **Description**: A CSV table that links each LULC code to specific parameters:

- USLE_C: Vegetation coverage and management factors. The cover-management factor from the Universal Soil Loss Equation (USLE). Unitless (0-1).

- USLE_P: This factor characterizes the inhibitory effect of human factors on soil erosion, ranging from 0 to 1. Among them, the P value of the area where soil erosion does not occur is 0, and the P value of the area where no protective measures have been taken is 1.

- SDR_RF: Retention efficiency factor for the LULC class. Unitless (0-1). Represents the trap efficiency of sediment before it reaches the flow path.

- SDR_MAX: Maximum retention efficiency for the LULC class. Unitless.

- **Sources:**

- USLE_C: Vegetation coverage and management factors (Wang et al., 1996)

- USLE_P: literature (Pan et al., 2010), Local land use planning documents.

| lucode | usle_c | usle_p |
| --- | --- | --- |
| 1 | 0.22 | 0.35 |
| 2 | 0.06 | 1 |
| 3 | 0.07 | 1 |
| 4 | 1 | 0 |
| 5 | 0.2 | 0 |
| 6 | 1 | 1 |

- **Format:** Table (.csv)
- **Watersheds**
- **Description**: Watershed boundaries are delineated from a depression-filled and peak-trimmed DEM by calculating flow direction and accumulation, extracting a river network using a threshold method, and identifying the basin outlet (Xu et al., 2004; Zhang et al., 2010).
- **Sources:** DEM: <https://www.gscloud.cn/>
- **Format:** Vector (.shp)
- **Threshold Flow Accumulation**
- **Description**: The number of upslope pixels that must flow into a pixel before it is classified as a stream.
- **Sources:** Literature values (Zhang et al., 2016), local monitoring data.
- **Format:** Number

Chen W., Xu J., Li S.. Water Conservation Function of Mountainous City in Western Yunnan: A Case Study of Baoshan City. Journal of Peking University (Natural Science Edition),2021,57(06):1153-1160.

Han H., Luo X., You R., et al. Analysis of water purification function in the Pearl River basin in Guizhou Province using InVEST model. Journal of Nanjing Forestry University (Natural Science Edition), 2016, 40(5): 87-92

Han J., Cui J., Yang W., et al. Analysis of Soil Erosion Change and Driving Factors in Low Hilly Areas Based on InVEST Model. Soil and water conservation research, 2022, 29 (5) : 32-39. DOI: 10.13869 / j.carol carroll nki RSWC. 2022.05.024.

Natural Capital Project, 2024. InVEST 3.14.1. Stanford University, University of Minnesota, Chinese Academy of Sciences, The Nature Conservancy, World Wildlife Fund, Stockholm Resilience Centre and the Royal Swedish Academy of Sciences.

Pan M., Wu Y., Ren F., et al. Estimating Soil Erosion in the Dongjiang River Basin Based on USLE. Journal of Natural Resources,2010,25(12):2154-2164.

Tang Z., Tian H., Wang Y., Zhang J., Li W., Wang T., Cheng J., Su C., Qi L. Scale effects of supplementary nature reserves on biodiversity conservation in China's southern hilly region. Journal of Environmental Management. 2025,123676.

Wang W., Jiao J. Qutantitative Evaluation on Factors Influencing Soil Erosion in China. Bulletin of Soil and Water Conservation,1996,(05):1-20.

Xu, X., Zhuang, D., Jia, S., Hu, Y. Automatic extraction method of river basin in China based on DEM in GIS environment. Resources and environment in the Yangtze River Basin. 2004,13(4),6.

Yan, F., Shangguan, W., Z., J. et al. Depth-to-bedrock map of China at a spatial resolution of 100 meters. Sci Data 2020,7, 2.

Zhang, G., Zhao, L., Xu, F., Xu, X. Watershed division scheme of China based on watershed structure analysis. Journal of Beijing Normal University (Natural Science Edition). 2010,46(03):417-423.

Zhang, X., Zhou, J., Li, M. Analysis of spatiotemporal changes in regional habitat quality based on land use pattern reconstruction. Acta Geographica Sinica. 2020,75(01), 160-178.

Zhang Y., Fan F., Zhou C., et al. Effects of Crop/Mulberry Intercropping on Surface Nitrogen and Phosphorus Losses in Three Gorges Reservoir Area. Acta Pedologica Sinica, 2016, 53(1): 189-201.

Zhou, W., Liu, G., Pan, J. et al. Distribution of available soil water capacity in China. J. Geogr. Sci. 15, 3–12 (2005).
